# Small molecule modulation of p75^NTR^ engages the autophagy-lysosomal pathway and reduces huntingtin aggregates in cellular and mouse models of Huntington's disease

**DOI:** 10.1016/j.neurot.2024.e00495

**Published:** 2024-11-26

**Authors:** Danielle A. Simmons, Namitha Alexander, Gloria Cao, Ido Rippin, Yarine Lugassy, Hagit Eldar-Finkelman, Frank M. Longo

**Affiliations:** a Department of Neurology & Neurological Sciences, Stanford University School of Medicine, Stanford, CA 94305, USA; b Wu Tsai Neurosciences Institute, Stanford University, Stanford, CA, USA; c Department of Human Molecular Genetics & Biochemistry, Faculty of Medicine and Health Sciences, Tel Aviv University, Israel

**Keywords:** Neurotrophin, Huntingtin inclusions, Neurodegeneration, Autophagy, p75^NTR^

## Abstract

Huntington's disease (HD) is a neurodegenerative disorder caused by a CAG repeat expansion in the *HTT* gene encoding a mutant huntingtin (mHtt) protein. mHtt aggregates within neurons causing degeneration primarily in the striatum. There is currently a need for disease-modifying treatments for HD. Many therapeutic studies have focused on lowering mHtt levels by reducing its production or enhancing its clearance. One way to clear mHtt aggregates is to promote autophagy, which is disrupted in HD. Our previous studies showed that the small molecule p75 neurotrophin receptor (p75^NTR^) ligand, LM11A-31, prevented HD-related neuropathologies and behavioral deficits in multiple HD mouse models. This study investigated whether modulating p75^NTR^ with LM11A-31, would reduce mHtt aggregates via autophagic/lysosomal mechanisms in HD models. LM11A-31 decreased mHtt aggregates in human neuroblastoma SH-SY5Y cells expressing mHtt (exon 1 with 74 CAG repeats) and in the striatum of R6/2 and zQ175dn mouse models of HD. The LM11A-31 associated decrease in mHtt aggregates *in vitro* was accompanied by increased autophagic/lysosomal activity as indicated by altered levels of relevant markers including p62/SQSTM1 and the lysosomal protease, mature cathepsin D, and increased autophagy flux. In R6/2 and/or zQ175dn striatum, LM11A-31 increased AMPK activation, normalized p62/SQSTM1 and LC3II levels, and enhanced LAMP1 and decreased LC3B association with mHtt. Thus, LM11A-31 reduces mHtt aggregates and may do so via engaging autophagy/lysosomal systems. LM11A-31 has successfully completed a Phase 2a clinical trial for mild-to-moderate Alzheimer's disease and our results here strengthen its potential as a candidate for HD clinical testing.

## Introduction

Huntington's Disease (HD) is a dominantly inherited neurodegenerative disorder characterized by motor, cognitive, and psychiatric symptoms mainly due to preferential degeneration of the striatum and eventual atrophy throughout the brain [1,2]. The disease is caused by a mutation in exon 1 of the *HTT* gene that extends the trinucleotide CAG repeat tract encoding a mutant huntingtin (mHtt) protein containing a polyglutamine expansion [3]. The expansion causes mHtt to aberrantly fold and aggregate in brain cells ultimately causing neuronal dysfunction and death [2,4]. Currently, disease-modifying treatments for HD do not exist and recent preclinical and clinical studies aiming to develop them have largely focused on therapies intended to decrease mHtt levels by reducing Htt production or enhancing mHtt clearance [4,5].

The main clearance process for long-lived, aggregate-prone proteins, including mHtt, that are too large for ubiquitin-proteosome degradation is macroautophagy (referred to as autophagy here), which is compromised in HD patient neurons and mouse models [[6], [7], [8], [9], [10]]. Wild-type (WT) Htt positively modulates selective autophagy which targets specific cargo, such as toxic proteins, for lysosome degradation [11]. WT Htt is present in the autophagy initiation complex and acts as a scaffold to enable binding of cellular components, including p62/SQSTM1 and LC3, needed for cargo recognition [10,11]. When mHtt is present, cargo recognition is impaired, autolysosome formation is disrupted, and mHtt is not efficiently degraded causing it to accumulate and aggregate in the cytoplasm and nucleus of cells ultimately causing their death [6,11,12]. Thus, increasing autophagic degradation of mHtt is a promising therapeutic target for HD and is supported by studies showing that enhancing autophagy in mouse and human neuron models of HD lowers mHtt aggregates [5,8,[13], [14], [15], [16], [17], [18], [19], [20]].

Autophagy may be modulated by signaling via the p75 neurotrophin receptor (p75^NTR^). p75^NTR^ is upstream of mammalian target of rapamycin (mTOR), a critical negative mediator of autophagy, and has been shown to regulate mTOR levels and phosphorylation during aging and degenerative conditions [10,[21], [22], [23], [24], [25]]. The mTOR inhibitor, rapamycin, has been shown to induce autophagy by activating p75^NTR^ signaling via the PTEN-AKT-mTOR pathway [21,22]. Independent of mTOR, p75^NTR^ interacts with tumor necrosis factor receptor-associated factor 6 (TRAF6) and p62/SQSTM1 which positively modulate autophagy [10,26]. LM11A-31 is an orally bioavailable small molecule ligand and modulator of p75^NTR^ that downregulates the receptor's degenerative signaling activity and upregulates its survival-promoting activity [27,28]. Our laboratory previously found that LM11A-31 reduced mHtt aggregates in the R6/2 mouse model of HD and elevated striatal PTEN levels. These changes were accompanied by reduced HD-related neuropathology, improved motor and cognitive ability, and a prolonged lifespan [27]. The reduction in mHtt aggregates may involve autophagic activity given the association between p75^NTR^ and autophagy-related signaling and machinery. This possibility was examined here by assessing the effects of modulating p75^NTR^ with LM11A-31 on autophagy-related processes and effector molecules using *in vitro* and *in vivo* HD models.

## Materials and Methods

### LM11A-31, a small molecule p75^NTR^ ligand

LM11A-31 is a water-soluble isoleucine derivative that has a structure similar to the loop 1 β-turn domain of NGF which interacts with p75^NTR^ [28,29]. The sulfate salt form of LM11A-31 [MW ​= ​439.34; (2*S*,3*S*)-2-amino-3-methyl-N-(2-morpholinoethyl) pentanamide] was used and custom manufactured by Ricerca Biosciences at >99 ​% purity. The MW of the free base is 243.35 (50 ​mg of the sulfate salt form of LM11A-31 contains 30 ​mg of the free base). The chemical structure and pharmacokinetics of LM11A-31 were previously published by our laboratory [28,30,31]. The brain half-life of LM11A-31 (50 ​mg/kg) is ∼3–4 ​h after a single dose and, after 2 weeks of once daily dosing, a peak brain tissue concentration of 463.4 ​ng/g was reached when assessed 30–60 ​min after the last dose [31]. The compound has completed a Phase 2a safety and exploratory endpoint clinical trial in mild-to-moderate Alzheimer's subjects [32].

### SH-SY5Y cell transfection with mHTT, immunoblotting, and autophagic flux assay

Human neuroblastoma SH-SY5Y cells were maintained in RPMI 1640/F12 medium supplemented with 10 ​% fetal calf serum (FCS), 5 ​mM l-glutamine, and 0.04 ​mg/ml gentamycin. Cells were transiently transfected with a GFP-mHtt plasmid that codes for the first exon of mHtt that harbors 74 CAG (Q) (kindly provided by Dr. David Rubinsztein University of Cambridge, UK) or GFP-Htt (23Q), which has a polyglutamine length in the WT range, using Lipofectamine 2000, according to the manufacturer's instructions (Invitrogen, Camarillo, CA, USA). Transfected cells were treated with LM11A-31 (200 ​nM) or vehicle (DMSO) for 16–18 ​h before they were either used for mHtt aggregate analysis or collected to prepare lysates for western immunoblotting. Cells used for mHtt aggregate analysis were grown on coverslips, ﬁxed with 4 ​% paraformaldehyde (PFA), washed with PBS, and mounted on glass slide with DAPI fluorescent mounting medium. Cells were visualized by confocal microscopy (Leica TCS-SP8) and the number of cells containing mHtt aggregates out of the total number of transfected cells ('green' cells) were counted manually from at least 20 randomly selected fields per experiment.

Cell lysates were prepared in an ice-cold buffer G (20 ​mM Tris-HCl, 10 ​% glycerol, 1 ​mM EDTA, 1 ​mM EGTA, 0.5 ​% Triton X-100, 0.5 ​mM orthovanadate, 10 ​mM β-glycerophosphate, 5 ​mM sodium pyrophosphate, 50 ​mM NaF, 1 ​mM benzaminidine, and the protease inhibitors aprotinin, leupeptin, and pepstatin (25 ​μg/ml each). Protein concentrations were determined by Bradford analysis and equal amounts of protein were subjected to gel electrophoresis followed by immunoblot analysis using methods similar to those described elsewhere [16]. Briefly, lysates were separated by electrophoresis on 10 ​% Bis-Tris gels then transferred onto nitrocellulose membranes. Membranes were probed using antibodies for WD repeat domain phosphoinositide interacting 2 (WIPI2; Abcam ab105459), p62/SQSTM1 (Sigma P0067), and cathepsin D (CatD; Santa Cruz Biotechnology sc-6486). GAPDH was used to show that equivalent levels of protein were loaded. All antibodies were used in a 1:500–1:1000 dilution range.

For the autophagic flux assay, SH-SY5Y cells expressing GFP-mHtt (74Q) were treated with LM11A-31 (200 ​nM) or DMSO for 18 ​h and then with chloroquine (CQ; 30 ​mM) or vehicle for 4 ​h. Cells were collected and lysates were prepared for LC3 immunoblotting, as described above, using 17 ​% Bis-Tris gels and anti-LC3B (Abcam ab192890).

### In vivo study design and dosing paradigms

This study aimed to determine the dose-response effects and mechanistic actions of the small molecule p75^NTR^ ligand, LM11A-31, on intranuclear mHtt aggregate reduction in male R6/2 and zQ175dn mice. R6/2 mice were utilized because they are useful for therapeutic testing since they develop HD symptoms rapidly and reliably [33,34]. Heterozygous zQ175dn mice were also used since they better represent the genetic component of HD and have a slower disease progression which allows targeting of early degenerative mechanisms [35,36]. The number of mice per group needed to obtain statistical significance was determined based on published studies with these two HD mouse models [27,35,36]. They were placed into groups based on body weight and age. Experimenters that were blind to the treatment and genotype conditions performed the dosing and histological and quantitative analyses.

Two cohorts of male R6/2 mice and their WT littermates were treated with vehicle or LM11A-31 (dissolved in distilled water GIBCO) starting at ∼4 weeks of age for 7 weeks. To investigate dose-response effects of LM11A-31, three doses of the salt form were used: 25, 50, and 100 ​mg/kg delivered via oral gavage (10 ​ml/kg) once daily 5–6 days/week (n ​= ​9–13 mice/group). Vehicle control groups received water in the same manner. The doses were chosen based on brain concentrations of LM11A-31 after oral delivery and biological effects determined in our previous *in vivo* studies, including those performed in R6/2 mice [27,28,31,37].

LM11A-31 was administered orally to two cohorts of male zQ175dn mice and their respective WTs for 4 months starting at 4.5–5 months of age at 4 non-zero concentrations *once or twice* daily (9 a.m. and 4 p.m.) 5 days a week. All mice received oral gavage twice a day; those receiving only one dose of compound per day were given vehicle (distilled water GIBCO) during their AM administration. Twice daily dosing was chosen to increase the ligand's brain exposure time as the half-life of LM11A-31 in mouse brain following a single oral-gavage dose of 50 ​mg/kg is ∼3–4 ​h [31]. Dosing twice a day was not feasible for R6/2 mice as the increased handling, which is known to induce seizures in this model, caused increased mortality [33,34]. The seven experimental groups were (n ​= ​7–9 mice/group): i) WT-Veh (vehicle given in the AM and PM); ii) zQ175-Veh (vehicle AM and PM); iii) zQ175-10 ​mg 2X [LM11A-31 (10 ​mg/kg) given in the AM and PM]; iv) zQ175-25mg 2X [LM11A-31 (25 ​mg/kg) AM and PM]; v) zQ175-50mg 1X [vehicle AM; LM11A-31 (50 ​mg/kg) PM]; vi) zQ175-50mg 2X [LM11A-31 (50 ​mg/kg) AM and PM]; and vii) WT-50 ​mg 2X [LM11A-31 (50 ​mg/kg) AM and PM].

### Mice and genotyping

All animal procedures were conducted in accordance with the National Institutes of Health Guide for the Care and Use of Laboratory Animals using protocols approved by the Institutional Animal Care and Use Committee at Stanford University. These protocols were designed to minimize animal suffering and numbers used. Mice were group-housed (3–5 mice/cage), except aggressors if fighting occurred, and received cotton nestlets and rodent chow *ad libitum*. Tail DNA was used for genotyping via real-time PCR and CAG repeat number measurement via ABI GeneMapper 4.0 by Laragen Inc (Culver City, CA). Male zQ175dn mice (JAX stock # 029928), and their WT littermates, and breeding pairs of R6/2 mice [female hemizygous ovarian transplant B6CBA-TgN (HD exon1)62, JAX stock #006494, and males from the same background strain] were purchased from Jackson (JAX) Laboratories. The zQ175dn knock-in mouse model encodes the human *HTT* exon 1 sequence with ∼190 CAG repeats inserted in place of the mouse *HTT* exon 1 and has had the floxed neo cassette removed (dn) since it can interfere with gene expression unrelated to HD [36]. R6/2 mice are transgenic for the 5′ end of the human *HTT* carrying 100–150 glutamine (CAG) repeats [33]. This study used male R6/2 mice that had an average of 128.3 ​± ​2.1 (mean ​± ​SD) CAG repeats.

### Immunohistochemistry procedures and quantification

Male R6/2 and zQ175dn mice, along with their respective WTs, were deeply anesthetized with sodium pentobarbitol 1 ​h after their last dose of LM11A-31 or vehicle. Next, they were transcardially perfused with saline solution and their brains were removed. One brain hemisphere was flash frozen and stored at −80 ​°C for western blotting while the other was immersion-fixed overnight in 4 ​% paraformaldehyde in 0.1 ​M phosphate buffer (PB; pH 7.4), cryoprotected in 30 ​% sucrose/PB, and sectioned (30 ​μm, coronal) using a freezing microtome. Free-floating sections were processed for immunohistochemical localization of aggregated Htt using the EM48 antibody [38] (1:300; clone EM48 made in mouse, MilliporeSigma, MAB5374), rabbit anti-p62 (1:500; MillporeSigma, P0067) or rabbit anti-phosphorylated acetyl CoA carboxylase (Ser79)(pACC; 1:500; Cell Signaling, 3661). These antibodies were visualized with the Vectastain Elite ABC kit (Vector Labs) and 0.05 ​% diaminobenzidine DAB (Sigma) in Tris-buffered saline (pH 7.5) with 0.03 ​% H_2_O_2_. Immunofluorescent staining for Htt (1:300; MilliporeSigma, MAB5374) combined with p62 (1:300; MillporeSigma, P0067) and rat anti-LAMP1 (1:200; DSHB, 1D4B), or LC3B (D11) XP® (1:200; Cell Signaling #3868) was also performed. As per the Cell Signaling datasheet, the specificity of the LC3B antibody was confirmed by the lack of signal in extracts from LC3B knockout cells (HCT 116) and stronger reactivity is observed with LC3II than LC3I. The secondary antibodies used were donkey anti-mouse AlexaFluor 488, donkey anti-rabbit AlexaFluor 647, or donkey anti-rat or anti-rabbit AlexaFluor 594 IgGs (all ThermoFisher Scientific 1:1000).

All immunostaining in the striatum was examined at rostral to mid-caudal levels [+1.18 to +0.02 ​mm relative to Bregma] with sample fields placed in the dorsolateral region, ventral to and abutting the corpus callosum. Intranuclear Htt staining in R6/2 mice was also examined in the primary motor cortex (+0.98 to +0.86 ​mm relative to Bregma) and dorsal hippocampus (−1.7 to −2.3 ​mm). Htt aggregates were evaluated by manually tracing the intranuclear inclusions in the striatum [sample field 250 ​× ​250 ​μm (62500 ​μm^2^), cortex [sample field (250 ​× ​250 ​μm) in cortical layers 4/5], and hippocampus [sample field (125 ​× ​25 ​μm) in CA1 pyramidal layer] in one section per mouse while viewing with a 100× oil objective of a Zeiss AxioImager M2 microscope. Aggregates containing p62/SQSTM1 and cell bodies with pACC immunostaining (2 sections/mouse) were analyzed the same way but only in the striatum (one sample field 250 ​× ​250 ​μm) of both R6/2 and zQ175dn mice. Tracings were performed using the “trace/contour mapping” command in Neurolucida v2021.1.1 (MBF) image analysis software while viewing live and focusing through the z-plane. The automated meander scan feature was used to prevent tracing overlapping fields. Htt and p62 immunostaining was performed in multiple sets, so quantifications were normalized to the R6/2- or zQ175-vehicle group of their respective cohorts.

Fluorescent double immunostaining for Htt (EM48) and either p62/SQSTM1, LAMP1 or LC3B was analyzed in two striatal sections of one brain hemisphere per mouse (*n* ​= ​4–6 mice/group). Sample fields in the dorsolateral striatum (for p62-Htt: 2 fields/section; for LAMP1-Htt: 4 fields/section; for LC3B-Htt: 2–3 fields/section) were imaged (z-stack: 0.2 ​μm step size, 26 steps) using the 63× oil objective of a Stellaris 5 confocal microscope (Leica Microsystems). The association between Htt and p62, LAMP1, or LC3B was quantified with Imaris X64 (ver 9.9.0; Bitplane AG) using background subtraction. For p62-Htt, the “colocalization” feature was used to assess the percent of intranuclear Htt immunostaining (diffuse and aggregated) above threshold colocalized with p62 immunostaining. For LAMP1-Htt and LC3B-Htt, the “surfaces” feature was used to define the diffuse and aggregated Htt immunostaining (filtered by size and sphericity), and then a separate “surface” channel was used to threshold the LAMP1 or LC3B immunostaining above the background. To evaluate the association between Htt and either LAMP1 or LC3B, a filter for the “surface” channels were used to measure the volume of LAMP1- or LC3B-immunopositive puncta within a 3.25 ​μm radius of nuclear Htt signal as autophagosomes and degrative lysosomes tend to accumulate in the perinuclear region.

### Western immunoblotting for striatal lysates from R6/2 mice

The striata from one brain hemisphere of R6/2 mice and their respective WT mice were prepared for Western blotting, as described previously [27]; zQ175dn lysates were used in another study. Briefly, brain tissue was homogenized by sonication in RIPA lysis buffer containing protease (cOmplete mini tablets, Roche cat. no. 11836153001) and phosphatase inhibitors. Protein concentrations were determined using Cytoskeleton Red Assay kit reagents (Cytoskeleton Inc.). For all western blotting procedures, tissue lysates from each genotype and treatment group were run on the same gel. For pACC immunoblotting, lysates were separated on NuPAGE 3–8% Tris – acetate gels with Tris-Acetate SDS running buffer (Invitrogen) containing NuPAGE anti-oxidant (Invitrogen; NP0005) and transferred to nitrocellulose membranes (ThermoFisher). Membranes were probed using rabbit anti-pACC (Ser79)(1:1000; Cell Signaling 3661) and mouse anti-vinculin (VCL) (1:500; Sigma V9131) as a loading control. For LC3B (microtubule-associated light chain 3B), tissue lysates were electrophoresed through NuPAGE 4–12 ​% Bis-Tris Gels with MES SDS running buffer (Invitrogen) and transferred to polyvinylidene difluoride membranes (PVDF Immobilon-FL, Millpore). PVDF membranes were probed using a monoclonal antibody for LC3B (D11) XP® (1:1000 Cell Signaling #3868, made in rabbit) and an α-tubulin antibody (Sigma, T6074) as a loading control. For all blots, secondary antibodies (IRDye® 800CW, IRDye® 680CW) were imaged with an Odyssey® CLx near-infrared fluorescence imaging system (Li-Cor Biosciences). Immunoreactive bands were outlined manually, background was subtracted, and band densities were measured using Image Studio Lite software (Li-Cor Biosciences). The densities of pACC and LC3II immunoreactive bands were expressed as a fraction of VCL or tubulin, respectively, in the same lane. Only LC3II was examined, rather than the commonly used LC3II to LC3I ratio, because LC3II is more sensitive to western immunoblotting detection and indicates autophagosome formation [39]. Samples were run in duplicate per mouse and data was normalized to the WT-Veh group of that gel and then averaged.

### Statistical analysis

Statistical analyses were conducted using GraphPad Prism (v.9.1) software. Statistical outliers were defined, *a priori*, as values that were two standard deviations from the mean and their removal, if needed, is noted in the figure captions. The number of mice and the statistical test(s) used for each analysis is also specified in the figure captions. First, data normality was determined using the Kolmogorov-Smirnov test; all data was normally distributed in this study precluding the need for non-parametric tests. When three or more groups were compared, the statistical significance of mean differences between normally distributed continuous variables with equal variances was determined using a one-way analysis of variance (ANOVA) with planned comparisons and a Fisher's LSD test. If two groups were compared, an unpaired *t*-test was used. Results are expressed as group mean ​± ​standard error of the mean (s.e.m.), and statistical significance was set at p ​≤ ​0.05.

## Results

### LM11A-31 reduces the number of cells with mHtt aggregates and increases autophagy markers and flux in vitro

**Fig. 1:**
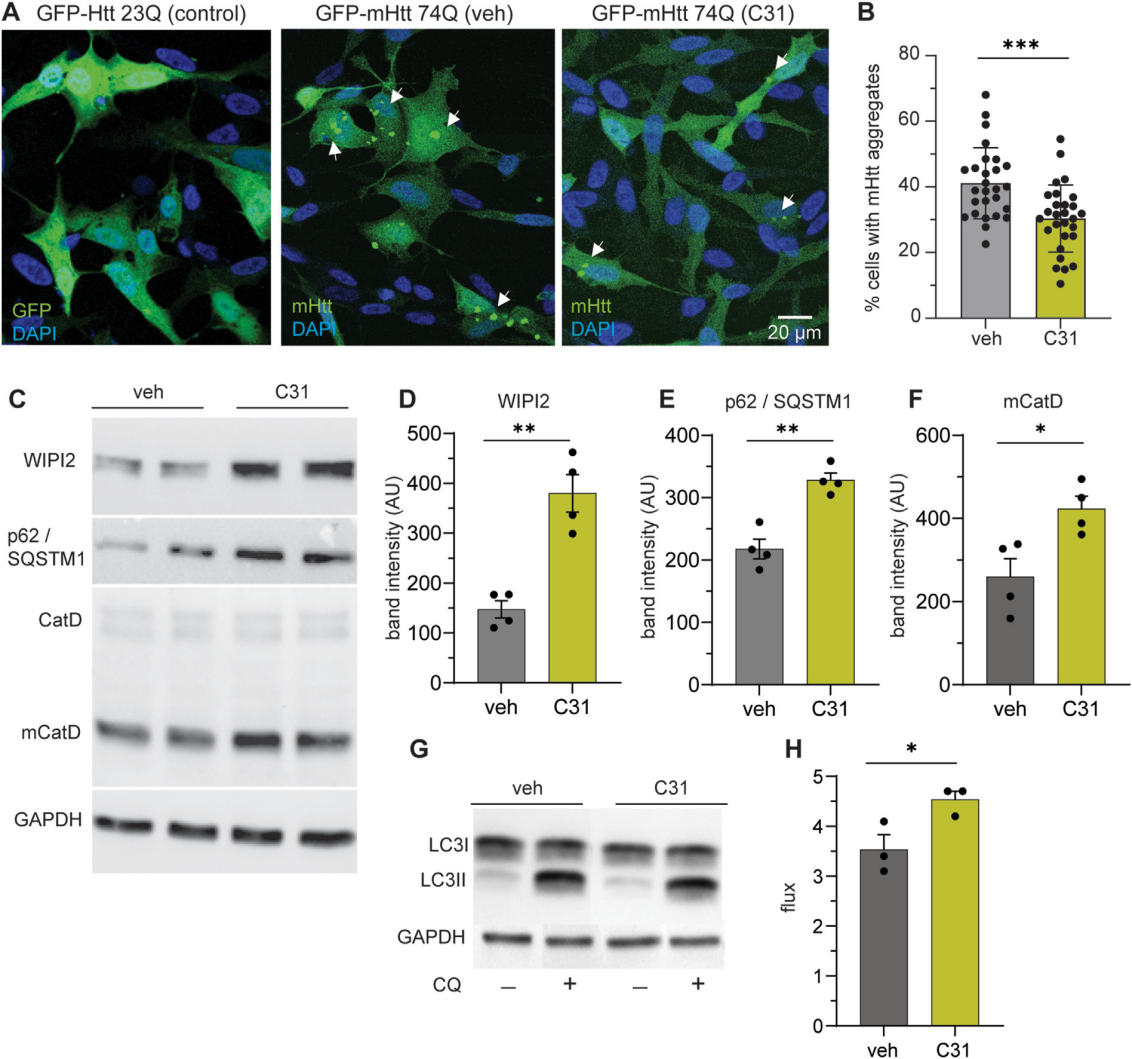
*LM11A-31 reduces mutant Htt aggregate-containing cells and increases autophagy markers and flux* in vitro. (**A**) Representative photomicrographs show SH-SY5Y cells expressing GFP-Htt (23Q) control or GFP-mHtt (74Q) that were treated with LM11A-31 (C31; 200 ​nM) or vehicle (veh; DMSO) for 16–18 ​h. Cells were fixed, stained with DAPI (blue), and visualized by fluorescence microscopy. White arrows point to mHtt aggregates in cells. (**B**) Bar graph showing the percentage of transfected cells containing mHtt aggregates. (**C**) Representative immunobands from western blots using lysates of SH-SY5Y cells expressing GFP-mHtt (74Q) treated with C31 or veh probing for the indicated autophagy markers: WD repeat domain, phosphoinositide interacting 2 (WIPI2), p62/SQSTM1, and cathepsin D (CatD) and its mature form (mCatD). The GAPDH immunoband is shown to demonstrate equal protein loading. (**D-F**) Bar graphs showing the densitometric analyses of the respective bands. AU ​= ​arbitrary units. (**G**) Representative immunobands from western blots using lysates of SH-SY5Y cells expressing GFP-mHtt (74Q) treated with chloroquine (CQ; +) or left untreated (−) followed by LM11A-31 or veh and probing for LC3II. The GAPDH immunoband is shown to demonstrate equal protein loading. (**H**) Bar graph showing autophagy flux as the ratio of LC3II in the absence (CQ-) and presence of CQ (CQ+). All results (mean ​± ​SEM) are expressed as fold change in relation to the veh-treated group. ∗p ​< ​0.05, ∗∗p ​< ​0.01, ∗∗∗p ​< ​0.001 by two-tailed t-tests (n ​= ​3 independent experiments).

Modulating p75^NTR^ with the small molecule ligand LM11A-31 decreased intranuclear mHtt aggregates in R6/2 mice [27]. Since p75^NTR^ is involved in autophagy, we investigated whether LM11A-31's effect on mHtt aggregates involves autophagic processes using an *in vitro* HD model. SH-SY5Y cells were transiently transfected with a GFP-mHtt [exon 1 with 74 CAG (Q) repeats] plasmid and then treated with LM11A-31 (200 ​nM) or vehicle (DMSO) for 16–18 ​h. As a control, other SH-SY5Y cells were transfected with GFP-Htt (23Q), which has a polyglutamine tract length in the WT range. We previously showed that the transfected 23Q plasmid did not produce Htt aggregates or up-regulate autophagy-related proteins in this cellular system [16]. Nuclear and cytoplasmic mHtt aggregates were abundant in the GFP-mHtt (74Q) transfected cells treated with vehicle but were not present in the GFP-Htt (23Q) transfected cells (Fig. 1A and B). Treatment with LM11A-31 reduced mHtt accumulation in the GFP-mHtt (74Q) expressing cells (Fig. 1A and B). This reduction in cells with mHtt aggregates was accompanied by significant alterations in typical autophagic markers as Western blotting revealed elevated levels of WIPI2, p62/SQSTM1, and mature cathepsin D (mCatD) in LM11A-31- versus vehicle-treated cells (Fig. 1C–F). WIPI2 is involved in autophagosome biogenesis, and mCatD is an aspartyl protease that participates in mHtt lysosomal degradation [[40], [41], [42]]. Elevated levels of WIPI2 and mCatD are indicators of enhanced autophagic activity. Acute increases in p62/SQSTM1 levels may facilitate autophagy and protect against mHtt toxicity, as indicated in a previous study using a similar HD cellular model [43].

To evaluate further evidence that LM11A-31 enhances autophagy, the effect of modulating p75^NTR^ on autophagic flux was assessed by treating GFP-mHtt (74Q) expressing SH-SY5Y cells with LM11A-31 followed by chloroquine (CQ). CQ is a lysosomotropic weak base that inhibits lysosome-mediated autophagic degradation [44]. This autophagic flux blockade causes LC3II accumulation when cells are exposed to autophagy-inducing conditions. This assay confirms that LM11A-31 increased autophagic flux, as measured by the ratio of LC3II levels in the presence or absence of CQ, compared to vehicle-treated cells (Fig. 1 G, H). The LM11A-31-induced increase in autophagic flux, in conjunction with increased WIPI2 and mCatD, strongly suggest that LM11A-31 enhances effective autophagy to decrease mHtt aggregates.

### LM11A-31 reduces intranuclear Htt aggregates in R6/2 and zQ175dn mice

**Fig. 2:**
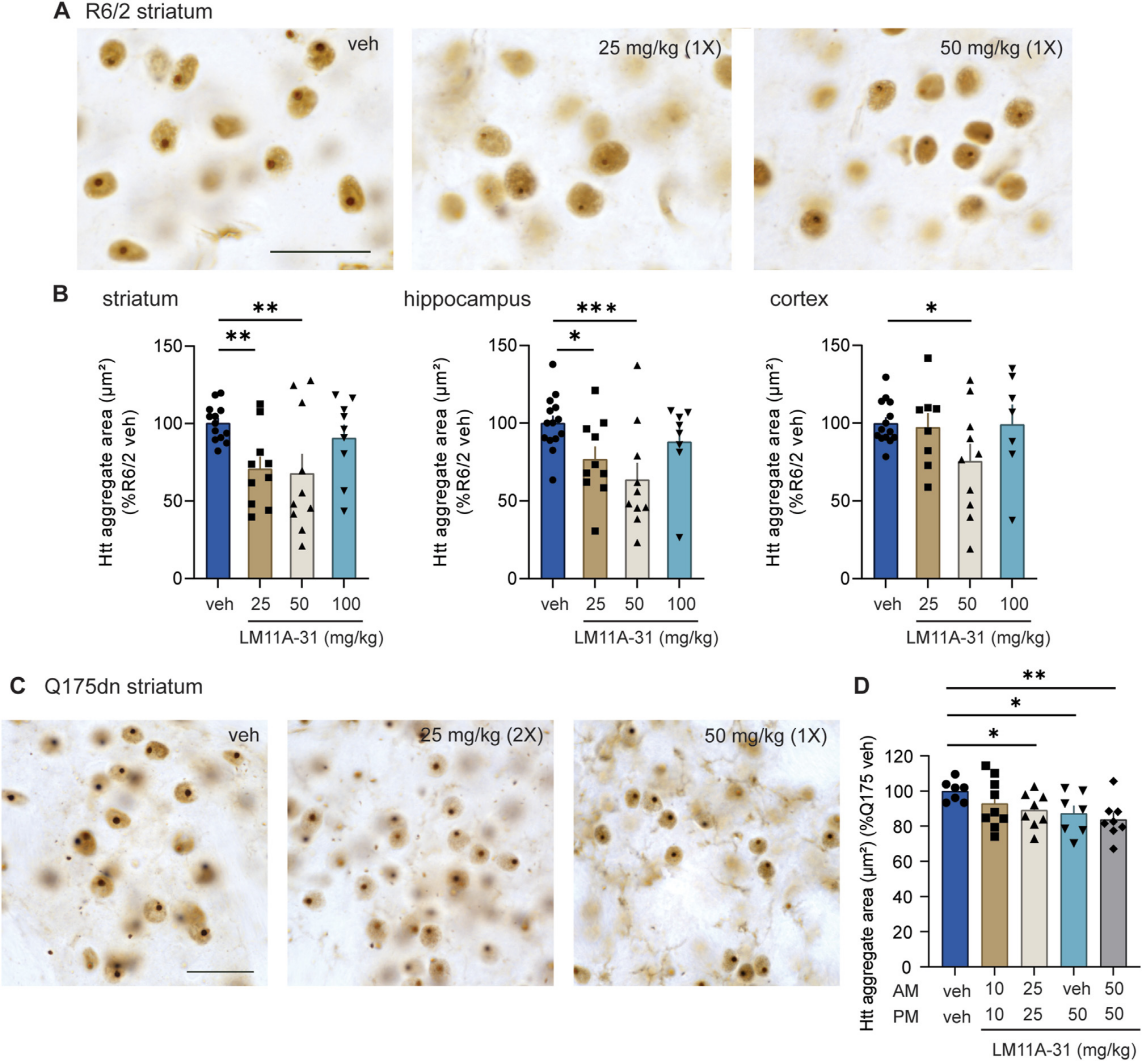
*LM11A-31 reduces intranuclear Htt aggregates in R6/2 and zQ175*dn *mice.* (**A**) Representative photomicrographs of Htt immunostaining (EM48) in the striatum of 11-week-old R6/2 mice given vehicle (veh, left panel) or once (1X) daily doses of LM11A-31 ​at 25 ​mg/kg (middle panel) or 50 ​mg/kg (right panel). Scale bar in A (left) ​= ​25 ​μm and applies to all panels in A. (**B**) Bar graphs showing the quantification of the percent area of intranuclear Htt aggregates (area analyzed: 62500 ​μm^2^) in the striatum, hippocampus, and cortex of R6/2 mice (*n* ​= ​8–13 mice/group). ∗p ​= ​0.03, ∗∗p ​≤ ​0.01, and ∗∗∗p ​≤ ​0.005 versus R6/2-veh group. (**C**) Representative photomicrographs of Htt immunostaining in the striatum of 9-month-old zQ175dn mice given veh (left panel) or LM11A-31 ​at 25 ​mg/kg twice daily (2X; middle panel) or 50 ​mg/kg once daily (1X; right panel). Scale bar in C (left) ​= ​25 ​μm and applies to all panels in C. (**D**) Bar graph showing the percent area of intranuclear Htt aggregates the zQ175dn striatum (area analyzed: 62500 ​μm^2^) (*n* ​= ​7–9 mice/group). ∗p ​≤ ​0.05 and ∗∗p ​≤ ​0.01 versus zQ175-veh group. All data was normally distributed, as established by the Kolmogorov-Smirnov test, and statistical significance was determined with an ANOVA and Fisher's LSD. All results (mean ​± ​SEM) are expressed as a percent of the HD-veh group for that mouse strain and immunostain.

WT Htt is diffusely located in the cytoplasm of cells however mHtt forms cytosolic aggregates and also migrates to the nucleus to form intranuclear inclusion bodies in numerous cell types and brain areas of HD subjects and mouse models [45]. mHtt aggregates begin to develop in R6/2 striatum, cortex, and hippocampus as early as 2–4 weeks of age [33,46]. We previously showed that modulating p75^NTR^ signaling with LM11A-31 (50 ​mg/kg) reduced the area of intranuclear mHtt aggregates in neurons of the striatum, cortex and hippocampus of male R6/2 mice [27]. In this study, we aimed to replicate our previous results and extend them to assess pharmacokinetic-pharmacodynamic relationships by including multiple doses of LM11A-31. Accordingly, the area of intranuclear mHtt aggregates was assessed in the striatum, cortex, and hippocampus of R6/2 mice at 11 weeks of age after oral gavage dosing for ∼7 weeks with vehicle (water) or LM11A-31 ​at doses of 25, 50, or 100 ​mg/kg. Compared to R6/2 mice given vehicle, we found that LM11A-31 reduced the area of mHtt aggregates in the striatum and hippocampus by ∼30 ​% when administered to R6/2 mice at doses of 25 and 50 ​mg/kg (Fig. 2A and B) and in the cortex by ∼25 ​% at the 50 ​mg/kg dose (Fig. 2B). LM11A-31 ​at 100 ​mg/kg did not affect mHtt aggregates in any brain area examined.

The dose-response effect of LM11A-31 on mHtt aggregates was also investigated in heterozygous zQ175dn mice since these knock-in mice have an HD mutation that most closely resembles humans in that they exhibit HD phenotypes with one WT and one m*HTT* allele [35,36]. Prominent aggregates emerge at about 4–6 months of age in heterozygous zQ175 mice, as detected by EM48 immunostaining, although they have been detected at 1–2 months of age using molecular assays [36,47,48]. A single 50 ​mg/kg dose of LM11A-31 administered to C57BL6/J mice by oral gavage reached peak brain concentrations 30 ​min post-dose and the brain half-life was ∼3–4 ​h [31]. This data suggests that dosing twice daily may increase brain exposure to LM11A-31 by maintaining levels between dosing bouts which may be more effective against neuropathology. This hypothesis was assessed by evaluating striatal mHtt aggregates after dosing zQ175dn mice once or twice daily (9 a.m. and 4 p.m.; 5 days/week; oral gavage) with vehicle or LM11A-31 starting at 4.5–5 months of age for 4 months. The LM11A-31 doses examined were 10, 25, and 50 ​mg/kg twice daily and 50 ​mg/kg once daily; mice receiving a single LM11A-31 dose per day were given vehicle for their morning dose. The area of intranuclear mHtt aggregates in the striatum was reduced by ∼10–15 ​% in zQ175dn mice given LM11A-31 ​at 25 and 50 ​mg/kg twice daily or 50 ​mg/kg once daily (Fig. 2C and D). This reduction was similar for all the doses suggesting that twice daily dosing was not more effective at reducing intranuclear aggregates than dosing once daily. LM11A-31 ​at 10 ​mg/kg twice daily did not affect mHtt aggregate area.

### LM11A-31 increases striatal AMPK activation as evidenced by elevated pACC levels

**Fig. 3:**
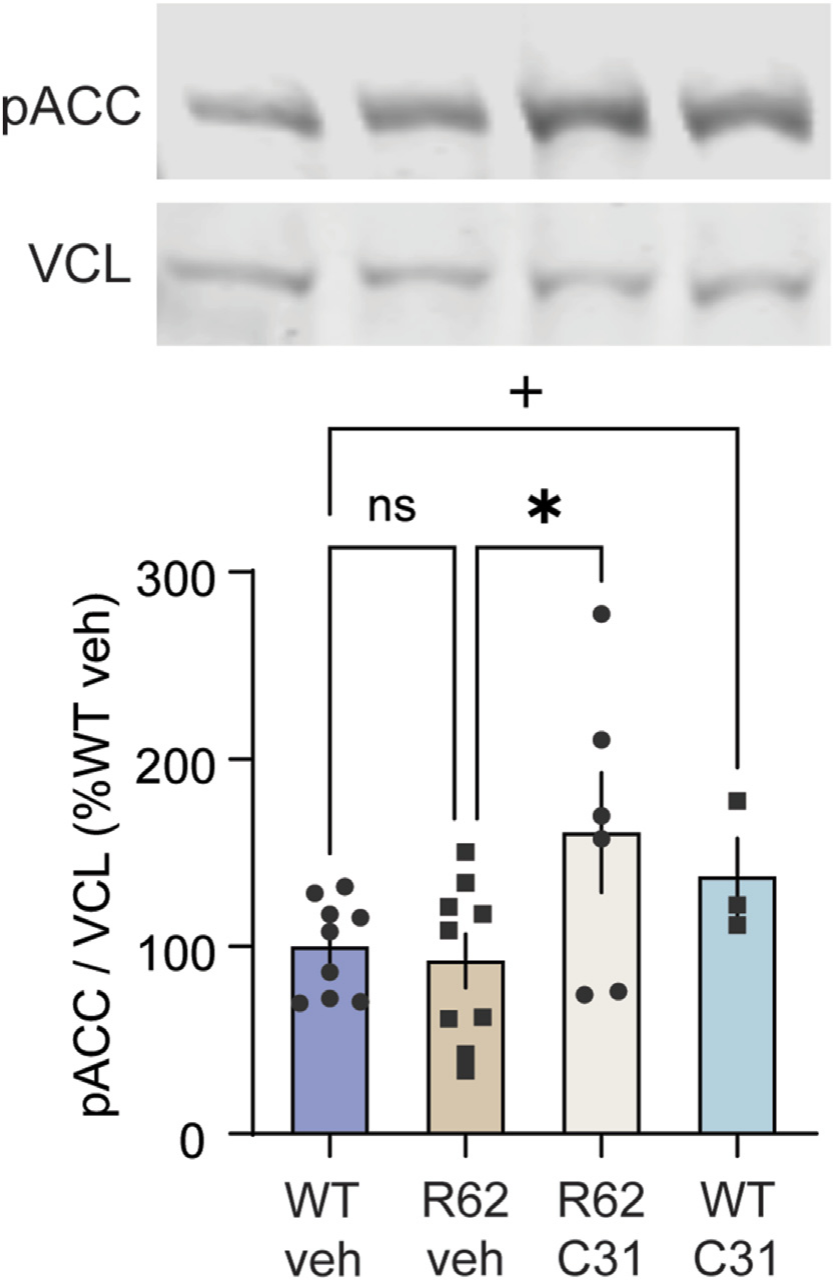
*LM11A-31 increases levels of pACC, an AMPK substrate, in the striatum of R6/2 mice.* Representative immunobands from western blots using striatal lysates from 11-week-old WT and R6/2 mice treated with vehicle or LM11A-31 (50 ​mg/kg once daily for ∼7 weeks) are shown. The bar graph depicts the results (means ​± ​SEM) of the densitometric analyses of the pACC immunobands from replicated western immunoblotting runs (2 runs/mouse; *n* ​= ​6–9 mice/group except WT-C31 *n* ​= ​3 mice) which are normalized to the WT-veh group run on the same gel. Vinculin from the same re-probed membrane is shown and was used as a loading control. Data is expressed as mean ​± ​SEM and is normally distributed, as determined by the Kolmogorov-Smirnov test. ns ​= ​not statistically significant; +p ​= ​0.036 versus WT-veh group (*t*-test); ∗p ​= ​0.01 versus R6/2-veh group (ANOVA, Fisher's LSD).

**Supplemental Fig. S1:**
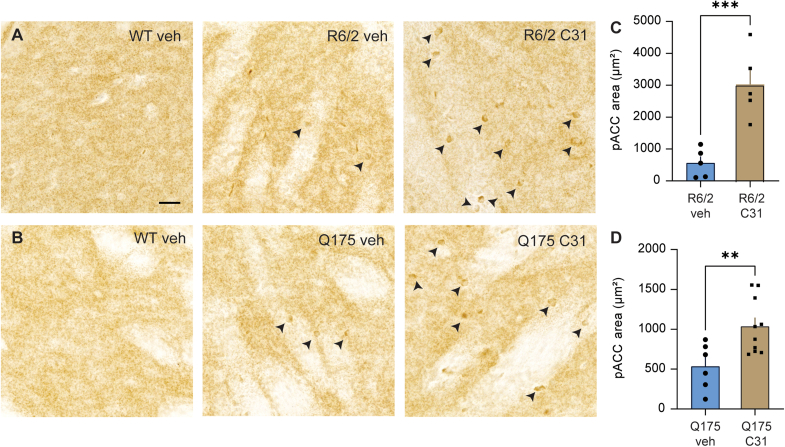
*LM11A-31 increases levels of pACC, an AMPK substrate, in the striatum of R6/2 and zQ175*dn *mice.* (**A, B**) Representative photomicrographs of pACC immunostaining in the striatum of 11-week-old WT and R6/2 mice given vehicle (veh) and an R6/2 mouse given LM11A-31 (50 ​mg/kg once daily) (**A**), and 9-month-old WT and zQ175dn mice given veh and a zQ175dn mouse given LM11A-31 (50 ​mg/kg once daily) (**B**). Arrowheads point to cellular pACC immunostaining distinct from the background. Note that few, if any, lightly immunostained-pACC cells were present in WT-veh and HD-veh mice. Scale bar in **A** (left) ​= ​20 ​μm and applies to all photomicrographs. (**C, D**) Bar graphs showing the quantification of the area of pACC immunostaining in the striatum of R6/2 mice (*n* ​= ​5 sections/group; 2-3 sections analyzed per mouse; *n* ​= ​2 mice/group) (**C**) and zQ175dn mice (*n* ​= ​6–10 sections/group; 2 sections/mouse; *n* ​= ​3–5 mice/group). Area analyzed per section: 62500 ​μm^2^. (**D**); ∗∗∗p ​= ​0.002 versus R6/2-veh group; ∗∗p ​= ​0.01 versus zQ175-veh group. All data is expressed as mean ​± ​SEM and is normally distributed, as indicated by the Kolmogorov-Smirnov test. Statistical significance was determined with two-tailed t-tests.

One potential mechanism for the decrease in mHtt aggregates seen with LM11A-31 is that its modulation of p75^NTR^ may instigate autophagic activity. Autophagy induction is facilitated by AMP-activated protein kinase (AMPK) via multiple routes including direct phosphorylation and activation of ULK1 and negative regulation of mTOR [49]. Similar to AMPK, p75^NTR^ is upstream of mTOR-related autophagic processes and can regulate mTOR levels and phosphorylation [21,22]. AMPK is activated by known autophagy inducers including metformin and rapamycin and has emerged as an HD therapeutic target as its activation via metformin or genetic up-regulation reduces mHtt aggregates and prevents neuronal loss in HD mouse models [17,[49], [50], [51], [52], [53]]. Thus, we investigated whether LM11A-31 affects AMPK activation by assessing Western immunoblotting for the standard AMPK substrate, pACC, in striatal lysates from R6/2 mice. Striatal pACC levels are low and do not differ between WT and R6/2 mice given vehicle, however they are elevated with LM11A-31 treatment in mice of both genotypes (Fig. 3). Immunostaining for pACC was also performed in R6/2 and zQ175dn mice. Diffuse punctate staining was seen throughout the striatum and WT and HD mice given vehicle had little, if any, light cellular pACC immunostaining, similar to the Western immunoblotting results showing low pACC levels in vehicle-treated mice. LM11A-31 increased the area of pACC immunostaining in the striatum of R6/2 and zQ175dn mice (Suppl. Fig. 1A and B). These results were similar to that seen with pACC immunostaining in zQ175 mice treated with metformin in a previous study [17]. Taken together these results suggest that LM11A-31 increases AMPK activation.

### LM11A-31 decreases intranuclear p62/SQSTM1 aggregates and their association with mHtt in R6/2 and zQ175dn striatum

**Fig. 4:**
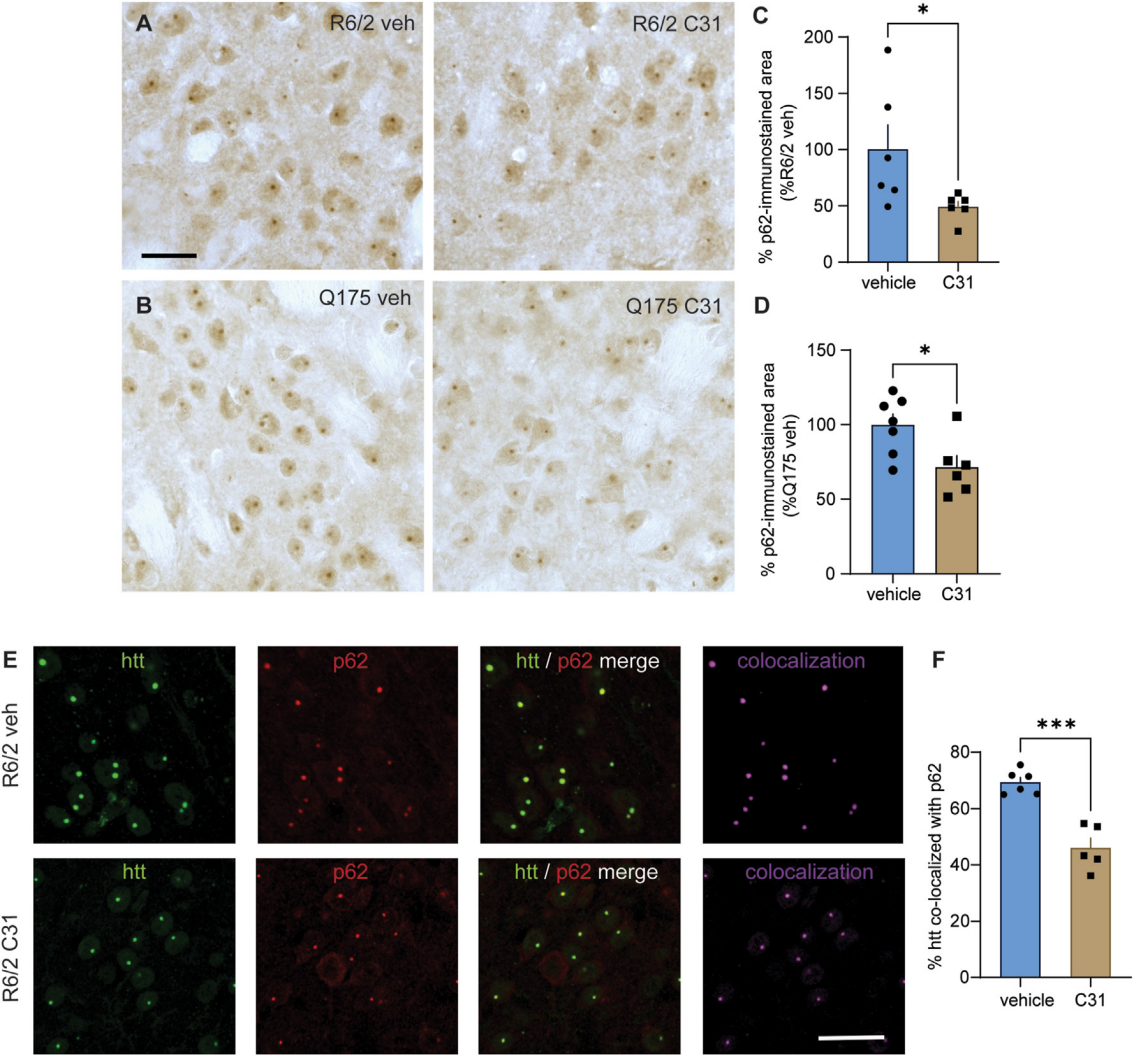
*LM11A-31 decreases intranuclear aggregates of p62/SQSTM1 and their co-localization with Htt in the striatum of HD mouse models.* (**A, B**) Representative photomicrographs of p62 immunostaining in the striatum of R6/2 mice (**A**) and zQ175dn mice (**B**) given vehicle (veh) or LM11A-31 (50 ​mg/kg once daily). Scale bar in **A** (upper left) ​= ​20 ​μm and applies to A and B. (**C, D**) Bar graphs showing the quantification of the percent area of p62-immunostained intranuclear aggregates in R6/2 mice (**C**) and zQ175dn mice (**D**); ∗p ​= ​0.044 versus R6/2-veh group (*n* ​= ​6 mice/group); ∗p ​= ​0.023 versus zQ175-veh group (*n* ​= ​6–7 mice/group). (**E**) Representative photomicrographs of Htt (htt, green) and p62 (red) fluorescent immunostaining and a rendering of their colocalization (purple) in the striatum of R6/2 mice given vehicle (veh) or LM11A-31 (50 ​mg/kg once daily). Scale bar in **E** (lower right) ​= ​10 ​μm. (**F**) Bar graphs showing the quantification of the percent of Htt co-localized with p62-immunostaining. ∗∗∗p ​= ​0.0002 versus R6/2-veh group (*n* ​= ​5–6 mice/group). All data is expressed as mean ​± ​SEM and is normally distributed, as determined by the Kolmogorov-Smirnov test. Statistical significance was determined with two-tailed t-tests.

Autophagy, when unimpeded, regulates mHtt levels and reduces intranuclear mHtt aggregates. This process involves the autophagy cargo receptor, p62/SQSTM1, which physically interacts with WT Htt to facilitate its association with LC3II leading to phagophore and autophagosome formation. Autophagosomes then fuse with lysosomes and p62/SQSTM1 is degraded [11,54]. mHtt impairs this process resulting in p62/SQSTM1 accumulation, which can be used as an indicator of autophagy status. Previous studies have shown that p62/SQSTM1 levels are unaltered in the striatum and cortex of R6/2 mice compared to WTs suggesting basal autophagy is not up-regulated [46]. When selective autophagy of protein aggregates, known as aggrephagy, is disrupted, p62/SQSTM1 accumulates in cortical and striatal neurons of R6/2 and zQ175 mice at 3 and 6 months of age, respectively, and is strongly associated with striatal Htt aggregates; this association and p62/SQSTM1 levels are decreased when autophagy is increased [15,17,46]. In this study, p62/SQSTM1 immunostaining was present diffusely in the nucleus and in intranuclear aggregates in the striatum of vehicle-treated 11-week-old R6/2 and 9-month-old zQ175dn mice (Fig. 4A and B). Compared to HD mice given vehicle, the area of the p62-immunostained aggregates was decreased in both mouse models after treatment with LM11A-31 (Fig. 4C and D). In striatum of R6/2-veh mice, a high percentage of aggregated Htt was colocalized with p62/SQSTM1 immunostaining; this association was significantly decreased in R6/2 mice given LM11A-31 (Fig. 4E and F; Pearson's coefficient for colocalized volume: 0.83 ​± ​0.02 R6/2-veh vs. 0.78 ​± ​0.03 R6/2-C31, p ​= ​0.04, *t*-test, n ​= ​5–6 mice/group). zQ175dn mice were not examined for this measure.

### LM11A-31 normalizes LC3II levels and reduces accumulation of LC3 near Htt aggregates

**Fig. 5:**
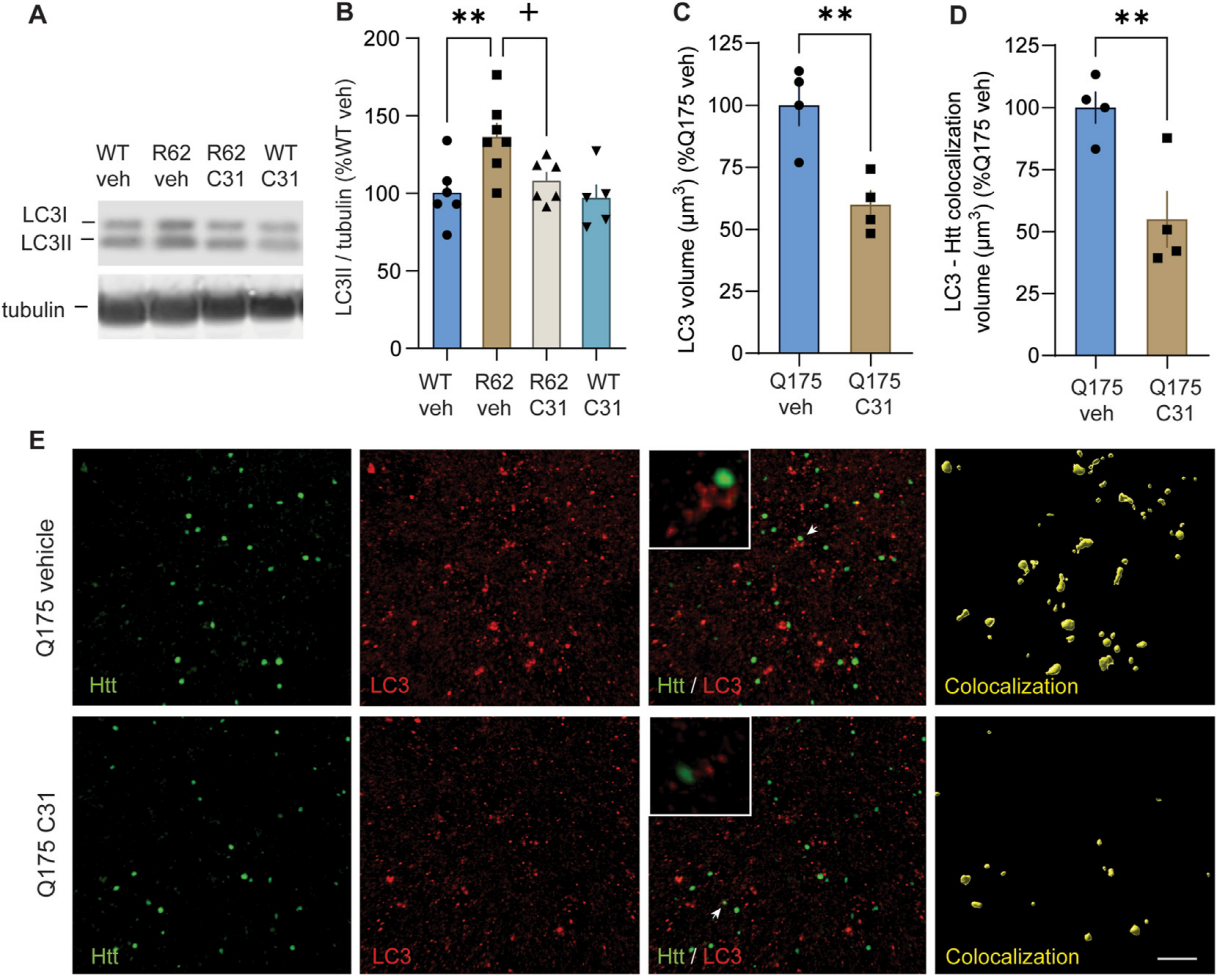
*LM11A-31 normalizes LC3II levels and decreases LC3 accumulation near Htt aggregates in the striatum of HD mice.* (**A**) Representative immunobands from western blots using striatal lysates from 11-week-old WT and R6/2 mice treated with vehicle or LM11A-31 (50 ​mg/kg once daily for ∼7 weeks) are shown. Tubulin from the same stripped and re-probed membrane is shown and was used as a loading control. (**B**) Bar graph depicting the results (means ​± ​SEM) of the densitometric analyses of the LC3II immunobands from replicated western immunoblotting runs (3 runs/mouse; *n* ​= ​5–7 mice/group) which are normalized to the WT-veh group run on the same gel. ∗∗p ​= ​0.004 versus WT-veh group; +p ​= ​0.02 versus R6/2-veh group (ANOVA and Fisher's LSD). (**C, D**) Bar graphs showing the quantification of the volume of LC3-immunostained puncta per field (**C**) and the association of these puncta with mHtt aggregates (**D**) in zQ175dn striatum. ∗∗p ​= ​0.004 (**C**) and ∗∗p ​= ​0.006 (**D**) versus zQ175dn-veh group (*n* ​= ​4 mice/group; t-tests). (**E**) Representative photomicrographs of Htt (green), LC3 (red), and their merged fluorescent immunostaining are shown along with a rendering of their colocalization (yellow) in the striatum of zQ175dn mice given vehicle (veh) or LM11A-31 (50 ​mg/kg once daily). Colocalization was defined as the volume of LC3-immunopositive puncta within a 3.25 ​μm radius of Htt signal. Inserts in **E** ​= ​10 ​μm width. The white arrows indicate the mHtt aggregate and LC3 shown at higher magnification in the inset. All data is expressed as mean ​± ​SEM and is normally distributed, as determined by the Kolmogorov-Smirnov test.

Next, we investigated whether LM11A-31 affects levels of LC3II, an active form of LC3 that correlates closely with autophagosome number [46,55]. Molecules linking mHtt to LC3 increase mHtt clearance and alleviate HD phenotypes in cell and *in vivo* models of HD [56]. LC3II is degraded by autophagy in autolysosomes and, if autophagosome-lysosome fusion is disrupted, LC3II accumulates, as seen in the R6/2 striatum [39,57]. Here, we show that striatal LC3II levels are elevated in R6/2-veh mice compared to WT-veh mice and that these levels are normalized in R6/2 mice given LM11A-31 (Fig. 5A and B). LM11A-31 did not affect LC3II levels in striatum of WT mice. The volume of LC3B-immunostained puncta and its accumulation near Htt aggregates is reduced in the striatum of zQ175dn mice with LM11A-31 treatment versus vehicle (Fig. 5C–E). These results suggest that dysfunctional autophagy leads to autophagosome/LC3II build-up in R6/2- and zQ175dn-veh mice and that LM11A-31 alleviates this aberrant accumulation.

### LM11A-31 enhances the lysosome marker LAMP1's association with Htt aggregates in R6/2 striatum

**Fig. 6:**
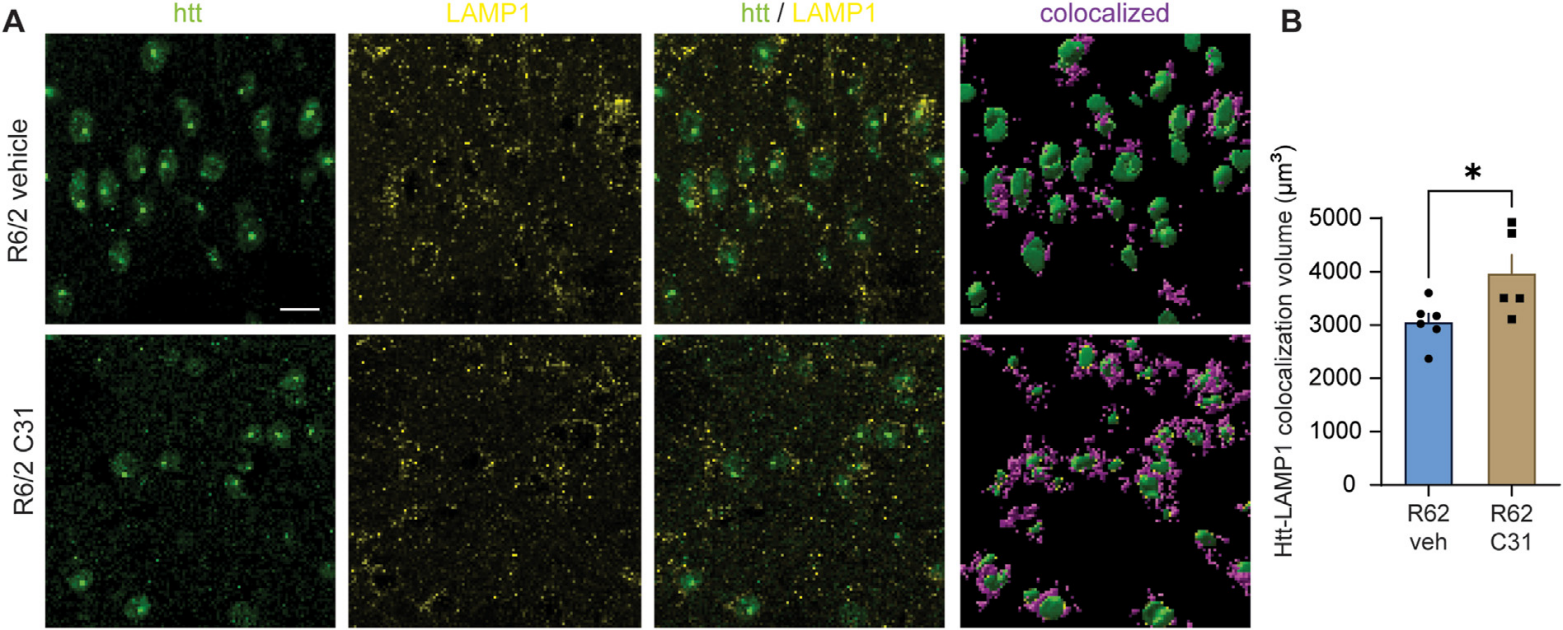
*LM11A-31 increases the association between intranuclear Htt and LAMP1 in R6/2 striatum*. (**A**) Representative photomicrographs of fluorescent immunostaining for huntingtin (htt, green), LAMP1 (yellow), and the merged channels as well as a rendering of their colocalization (purple) in the striatum of R6/2 mice given vehicle (veh) or LM11A-31 (50 ​mg/kg once daily). Scale bar in **A** (upper left) ​= ​10 ​μm and applies to all panels. (**B**) Quantification of the volume of diffuse and aggregated intranuclear Htt immunostaining colocalized with LAMP1 immunostaining. Colocalization was defined as the volume of LAMP1-immunopositive puncta within a 3.25 ​μm radius of Htt signal. ∗p ​= ​0.04 versus R6/2-veh group (*n* ​= ​5–6 mice/group). All data is expressed as mean ​± ​SEM and is normally distributed, as determined by the Kolmogorov-Smirnov test. Statistical significance was determined with a two-tailed *t*-test.

LC3II interacts with p62/SQSTM1 to bind to autophagosomes, which fuse with lysosomes to degrade cargo, in this case mHtt. We examined the degree to which intranuclear Htt was associated with the lysosomal membrane protein, LAMP1, following LM11A-31 treatment, using immunostaining for subcellular localization. The proximity of LAMP1-immunstained puncta to intranuclear mHtt immunostaining was used to investigate this association, rather than direct co-localization, as degradative lysosomes are largely concentrated in perinuclear regions and we would expect Htt directly co-localized with lysosomes to be rapidly degraded. R6/2 mice treated with LM11A-31 showed an increase in perinuclear LAMP1 in cells with mHtt compared to R6/2-veh mice (Fig. 6). This finding is consistent with an LM11A-31-mediated increase in mHtt-lysosome interactions likely involving autophagic activity as mHtt aggregate area was reduced.

## Discussion

**Fig. 7:**
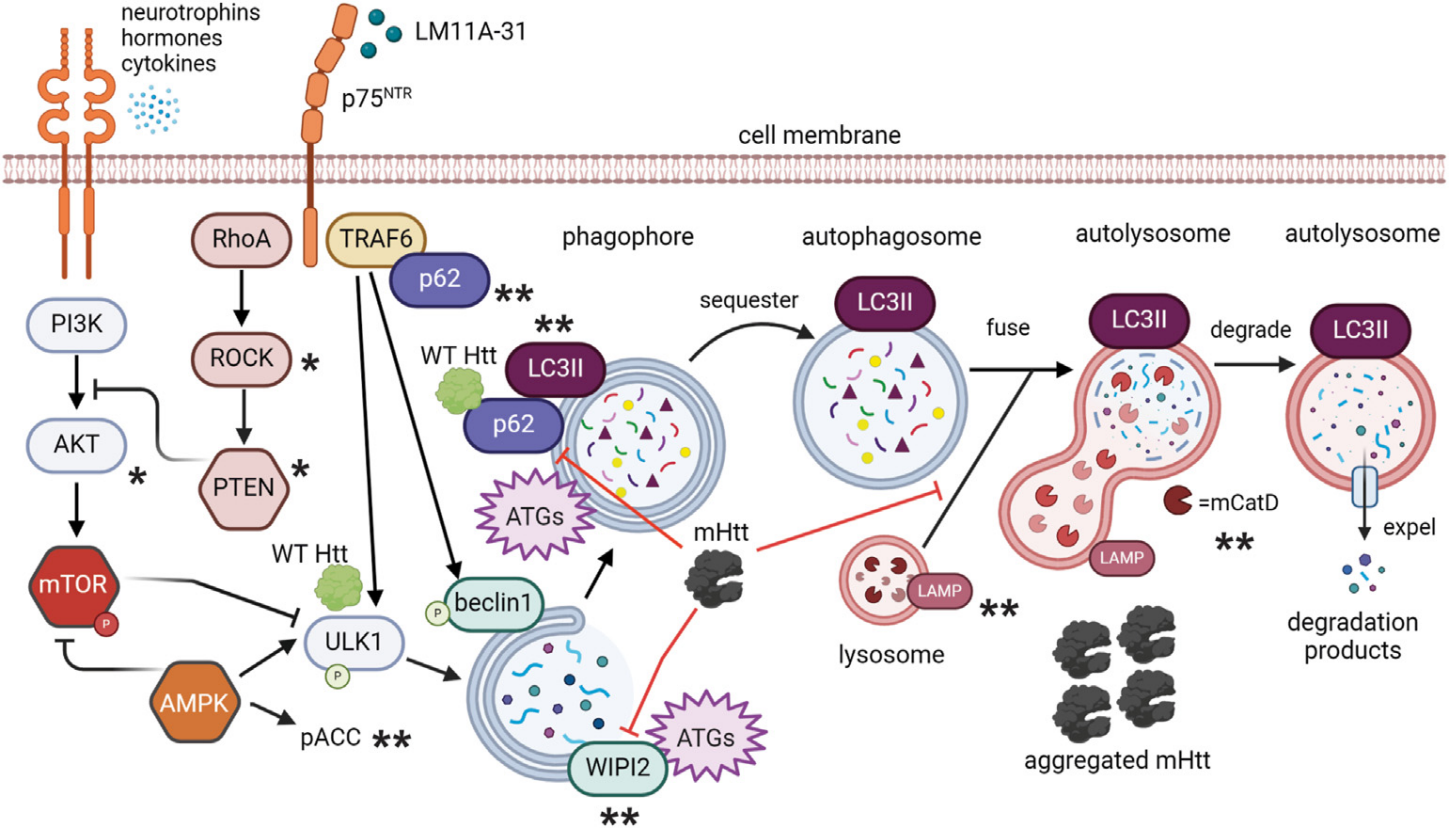
*Mechanistic model of p75*^*NTR*^*modulation of the autophagy-lysosomal pathway to reduce mHtt aggregates: effects of LM11A-31.* A schematic diagram showing potential mechanisms of p75^NTR^ involvement in the autophagy-lysosomal pathway and some of the ways that mHtt disrupts this process (red lines) leading to aggregation of the uncleared protein. If autophagy is functioning normally, mHtt would be recognized by the p62/SQSTM1 and LC3 complex and captured by the autophagosome for degradation. The small molecule p75^NTR^ ligand, LM11A-31, affects signaling intermediates and autophagic machinery critical for clearing mHtt aggregates at multiple stages of the process. Positively regulating autophagy at multiple steps may have high therapeutic value especially at the initiation stages. Asterisks indicate LM11A-31's effects in HD models that are described in the current study (∗∗) and in previously published work (∗) [27].

This study showed that modulating p75^NTR^ with LM11A-31 reduced mHtt aggregates in cellular and mouse models of HD and that this reduction was accompanied by molecular changes suggesting increased autophagic and lysosomal activity. Defective proteostasis is a common feature of many neurodegenerative diseases, including Alzheimer's, Parkinson's and HD, and leads to intracellular and intranuclear aggregation of misfolded proteins which can be toxic if they are not cleared [9,10]. The main clearance system is autophagy, up-regulation of which has been suggested as a therapeutic target for neurodegenerative diseases. As depicted in Fig. 7, autophagy, including aggrephagy, may be initiated via AMPK's activation of ULK1 or negative regulation of mTOR; mTOR may also be disinhibited by blocking the AKT pathway [9,18,50]. Other mTOR-independent pathways that may instigate the process include TRAF6-related signaling [26,50,58]. The autophagic process entails several steps starting with phagophore biogenesis, which requires various autophagy-related proteins (ATGs) and WIPI2 for membrane extension [9,42]. LC3II and p62/SQSTM1 are recruited, with WT Htt acting as a scaffold, forming autophagosomes to capture bulk cytoplasmic contents for basal autophagy, and in the case of aggrephagy, aggregated proteins such as mHtt [9,11,54]. Next, autophagosomes fuse with lysosomes to form autolysosomes, that contain acidic lysosomal proteases, such as mCatD, which degrade cargo and release the degraded products to maintain cellular homeostasis in constant basal autophagy, and to clear misfolded/aggregated proteins in aggrephagy [9,10,41]. In HD, basal autophagy is unperturbed in R6/2 mice but aggrephagy is disrupted at multiple phases of the process, including autophagosome formation and cargo recognition involving p62/SQSTM1 in HD patient neurons and mice [6,8,12,18,46]. Thus, HD treatments aimed at regulating this system may have higher therapeutic value if they target and modulate several of these steps.

LM11A-31 stimulated molecular changes that occur with increased autophagy at multiple steps in the aggregate clearing process. These changes involve the initial biochemical induction phases of autophagy as LM11A-31 increased pACC, an indicator of AMPK activation [17,50,51]. AMPK signaling is one of the main pathways regulating autophagy at multiple steps in the process including initiation, by activating ULK1 and negatively regulating mTOR, and promotes autophagosome formation [49]. We previously showed that LM11A-31 increased PTEN levels in R6/2 striatum which could initiate autophagic activity via another route by blocking the Akt pathway to remove mTOR inhibition of autophagy [27](Fig. 7). LM11A-31 also increased autophagic flux, elevated WIPI2, and altered p62/SQSTM1 in directions that may indicate facilitation of middle stages of the process during which autophagosomes form and cargo recognition occurs [10,12,42]. Finally, LM11A-31 increased the association between mHtt and lysosomes and elevated mCatD which is critical for the final degradation steps needed for aggregate clearance [10,15,41,59](Fig. 7). These results are similar to those reported in other preclinical studies that investigated HD treatments to induce autophagy and lower mHtt aggregates including those using L807mts to inhibit GSK-3 (glycogen synthase kinase-3), SAFit2 to inhibit FKFB5 (FK506 binding protein 5), ginkgolic acid to inhibit SUMOylation, metformin to activate AMPK, and rapamycin or trehalose to inhibit mTOR [5,[14], [15], [16], [17],^19^,^20^]. Some of these have also been shown to improve cognition in HD patients including metformin and rapamycin [9,18,52]. Together these studies indicate that autophagy up-regulation may be an effective intervention for mHtt aggregate clearance in HD.

LM11A-31's engagement of the autophagy/lysosomal system was accompanied by reduced mHtt aggregates in a cellular and two mouse models of HD. Examination of the dose-response effects of LM11A-31 on mHtt aggregate formation revealed that in the zQ175dn mouse model the three highest doses of LM11A-31, but not the lowest dose, significantly reduced the area of aggregates with a maximum effect at 25 ​mg/kg twice daily. These results indicate that a dose-response effect was present and a dose plateau was reached at the higher doses. In the R6/2 model, we replicated our previous finding that mHtt aggregates were reduced with LM11A-31 (50 ​mg/kg once daily) in the striatum, hippocampus and cortex. An atypical dose-response effect was seen in that the two lowest doses (25 and 50 ​mg/kg once daily) of LM11A-31 reduced aggregate area while the highest dose (100 ​mg/kg once daily) did not. Many factors can contribute to such an effect, for example, the high dose could have triggered p75^NTR^ to recruit one of its additional co-receptors which may have inhibited the signaling necessary to reduce the mHtt aggregates. p75^NTR^ levels are increased in the striatum and hippocampus of HD patients and multiple HD mouse models, including R6/2 mice, compared to healthy controls, but to a lesser extent or not at all in the cortex [27,60,61]. Interestingly, this regional pattern of increased p75^NTR^ expression parallels the effects of the p75^NTR^ ligand on mHtt aggregates in that C31's reduction in aggregate area was not as robust in the cortex as it was in the striatum and hippocampus, where p75^NTR^ levels are higher in the disease state.

The close association between mHtt and p62/SQSTM1 and the sequestration of p62/SQSTM1 into mHtt-containing aggregates may suggest that the decrease in intranuclear p62/SQSTM1 levels seen with LM11A-31 in R6/2 and zQ175dn striatum could be a consequence of reducing mHtt aggregates rather than autophagy initiation. However, LM11A-31 increased p62/SQSTM1 levels along with other autophagy markers and increased autophagic flux in ‘neuron like’ human neuroblastoma cells containing mHtt indicating autophagic activity. The opposing directions of LM11A-31's effects on p62/SQSTM1 in *in vitro* and *in vivo* HD models can be explained by the differing cellular context in which autophagy is stimulated. An increase in p62/SQSTM1 levels would be expected with acute stimulation of autophagy in cells in which the molecular machinery needed for the autophagic process has not been compromised by long-term exposure to toxic proteins including mHtt or by other means. This situation parallels the conditions seen in our *in vitro* experiments in which p62/SQSTM1 levels were increased in neuroblastoma cells transiently transfected with mHtt after acute LM11A-31 treatment. Increased p62/SQSTM1 levels have been shown in HD human fibroblasts and other *in vitro* HD models following treatment with autophagy inducers including amino acid starvation and rapamycin and may facilitate autophagy to protect against mHtt toxicity [12,15,43,54,62]. Up-regulating p62/SQSTM1 and basal, selective autophagy can degrade polyglutamine aggregates and prolong the lifespan of *C. elegans* [62]. When the autophagic process is intact, p62/SQSTM1 physically interacts with ubiquinated cargo such as mHtt aggregates and binds directly to LC3II leading to autophagosome-lysosome fusion and cargo degradation accompanied by p62/SQSTM1 itself being rapidly degraded [11,54]. In HD, this process is disrupted causing p62/SQSTM1 to accumulate and to be enveloped into intranuclear aggregates, which indicates inhibited autophagosome synthesis or impaired autophagic degradation [7]. Thus, LM11A-31's reduction of p62/SQSTM1 and its association with intranuclear mHtt aggregates in HD mice, along with its decreasing of aggregates, is consistent with the small molecule aiding clearance of mHtt potentially before it translocates to the nucleus and aggregates. The latter possibility is supported by the increased perinuclear LAMP1, a marker for lysosomes, in cells with mHtt in the R6/2 striatum and elevated mCatD levels seen *in vitro* with LM11A-31 treatment. LM11A-31 could also lower mHtt expression leading to reduced aggregates and this might occur via the autophagy/lysosomal systems.

The results presented in this study offer further understanding of p75^NTR^'s role in the autophagy-lysosomal pathway in that, in addition to neuroprotective effects, it may engage this pathway to remove aggregated proteins. Previous studies demonstrated that p75^NTR^ can regulate autophagic activity to provide neuroprotection during aging and degenerative conditions [[21], [22], [23], [24], [25]]. Silencing the p75^NTR^ gene (NGFR) reduces pro-autophagic markers in multiple cell types, including neurons, and this effect was associated with mTOR-related autophagic processes [21,24,63]. Rapamycin initiates autophagy at least in part by transactivating the p75^NTR^ promoter and activating p75^NTR^ downstream signaling via the PTEN/PI3K/Akt/mTOR pathway affecting cell viability [21,22]. Similarly, modulating p75^NTR^ signaling with LM11A-31 in mouse models of HD is neuroprotective and improves longevity [27]. Here, we show that it also increases autophagic activity which likely plays a major role in clearing mHtt and degrading mHtt aggregates, although other clearance mechanisms, such as those involving the proteosome, may also be involved. Interestingly, the effect of LM11A-31 on aggregate reduction may be preferential for mHtt as we showed previously that it does not affect accumulation of another β-sheet forming protein, amyloid-β, in Alzheimer's disease mouse models [31,64]. This study used male R6/2 and zQ175dn mice and while to our knowledge sex differences in the autophagy process have not been reported and we showed previously that LM11A-31 prevented striatal atrophy and neuroinflammation in male and female R6/2 mice [65], future studies should investigate the effects of LM11A-31 on autophagy in both male and female mice.

In conclusion, our results demonstrate that the small molecule p75^NTR^ ligand, LM11A-31, reduces mHtt aggregates and may do so by engaging the autophagy-lysosomal pathway. Up-regulating autophagic clearance of aggregated proteins, including mHtt, may be a safer alternative to Htt lowering therapies targeting huntingtin DNA and RNA as many of these also affect WT Htt. Given that LM11A-31 has been shown to reduce HD phenotypes in multiple mouse models and has successfully completed a Phase 2a clinical trial for mild-to-moderate Alzheimer's disease [32], it may be a prime candidate for HD clinical proof-of-concept testing.

## Author Contributions

The research was designed by D.A.S, H.E., and F.M.L.; D.A.S, G.C., N.A., Y.L., and I.R. performed the research and analyzed data; D.A.S. wrote the manuscript and H.E. and F.M.L. critically revised it.

## Declaration of competing interest

The authors declare the following financial interests/personal relationships which may be considered as potential competing interests: Dr. Frank M. Longo is listed as an inventor on patents related to LM11A-31 that are assigned to the University of North Carolina, University of California (UC), San Francisco and the Department of Veterans Affairs (VA). He is also entitled to royalties distributed by UC and the VA per their standard agreements. Dr. Longo is a principal of, and has financial interest in, PharmatrophiX, a company focused on the development of small-molecule ligands for neurotrophin receptors, which has licensing of several related patents. The other authors declare that they have no known competing financial interests or personal relationships that could have appeared to influence the work reported in this paper.
